# 3D Molecular Modelling Study of the H7N9 RNA-Dependent RNA Polymerase as an Emerging Pharmacological Target

**DOI:** 10.1155/2013/645348

**Published:** 2013-09-25

**Authors:** Dimitrios Vlachakis, Argiro Karozou, Sophia Kossida

**Affiliations:** Bioinformatics & Medical Informatics Team, Biomedical Research Foundation, Academy of Athens, Soranou Efessiou 4, 11527 Athens, Greece

## Abstract

Currently not much is known about the H7N9 strain, and this is the major drawback for a scientific strategy to tackle this virus. Herein, the 3D complex structure of the H7N9 RNA-dependent RNA polymerase has been established using a repertoire of molecular modelling techniques including homology modelling, molecular docking, and molecular dynamics simulations. Strikingly, it was found that the oligonucleotide cleft and tunnel in the H7N9 RNA-dependent RNA polymerase are structurally very similar to the corresponding region on the hepatitis C virus RNA-dependent RNA polymerase crystal structure. A direct comparison and a 3D postdynamics analysis of the 3D complex of the H7N9 RNA-dependent RNA polymerase provide invaluable clues and insight regarding the role and mode of action of a series of interacting residues on the latter enzyme. Our study provides a novel and efficiently intergraded platform with structural insights for the H7N9 RNA-dependent RNA Polymerase. We propose that future use and exploitation of these insights may prove invaluable in the fight against this lethal, ongoing epidemic.

## 1. Introduction

H7N9 is a serotype of the species *Influenzavirus A* that causes influenza in birds. *Influenzavirus A*, which is an enveloped virus, belongs to the family of Orthomyxoviridae, and kills more than 250,000 people worldwide every year on average. Its genome is comprised of eight negative sense, single-stranded RNA segments that encode eleven RNA proteins. A further classification of the influenza virus is based on two glycoproteins of its surface: the hemagglutinin (H) and the neuraminidase (N). There are 17 different hemagglutinin subtypes and 10 different neuraminidase subtypes.

In February 2013, the novel avian-origin influenza A (H7N9) virus that emerged in Eastern China. H7N9 was first reported to have infected humans [1, 2]. The World Health Organization (WHO) was notified of illness onset between February 19 and March 15, 2013, when three human cases of influenza A (H7N9) were confirmed in Shanghai and Anhui. Five to ten days later, the patients developed severe pneumonia and progressive respiratory distress with lethal outcome. As of July 4, 2013, 133 documented human cases were confirmed and 43 cases ended in death (human infection with avian influenza A (H7N9) virus, 2013). Available evidence suggests that most people have been infected after having contact with infected poultry or contaminated environments [3].

Evolution of influenza viruses is mainly based on mutations and reassortments [4]. RNA segments frequently reassort when the same host is infected by up to one strain of virus, a process that is favored by the segmented nature of the genome [5]. In addition, the mutation rate of viral genome is high during replication since the viral RNA-dependent RNA polymerase (RdRp) lacks proofreading ability [4]. 

H7N9 virus is one subgroup among the larger group of H7 viruses, which normally circulate among birds. A few isolated cases of human infection with H7 influenza viruses were reported in The Netherlands, Italy, Canada, United States of America, Mexico, and the United Kingdom between 1996 and 2012 (H7N2, H7N3, H9N2, or H10N7) [6–11]. They were low pathogenic and caused lower respiratory tract illness that was mild to moderate in severity with the exception of one death, which occurred in The Netherlands [8, 12]. The transmission of H7 viruses to mammals has been reported only rarely [13] in Asia, while human infections with N9 subtype viruses had not been referred anywhere.

H7N9 carries genes from rare H9N2, H7N3, H4N9, and H11N9 bird flu viruses [14]. The six internal genes are derived from influenza A (H9N2) viruses circulating in birds in eastern Asia. The HA gene is most closely related to duck H7N3 viruses but shares only ~95% identity. H4N9 and H11N9 isolated viruses showed considerable similarity to the NA genes from H7N9 viruses detected in birds [15]. The origins of their HA and NA genes remain unclear. 

Genes of H7N9 virus also show signs of adaption to the growth in mammalians. To acquire the potential to infect humans, these avian viruses evolve a binding affinity of HA for the a −2,6 linkage, which is preferentially involved in the mutations in the RBS regions of HA. A variety of surveys [16–19] have been conducted regarding H7N9 binding to mammalian cell receptors.

Ongoing outbreak in China of avian influenza related to highly pathogenic forms of the human virus has highlighted the urgent need for new effective drug development approaches. H7N9 strain is the most lethal strain of influenza viruses [1, 2]. Present medications and vaccines seem unfeasible to alleviate greatly an epidemic, while the risk of a pandemic is claimed to be very real due to H7N9 virus resistance in antivirals [20, 21]. Oseltamivir and zanamivir are sialic acid-mimicking inhibitors of NA [22] and are developed by structure-based drug design efforts [23], but resistant influenza is already emerging [24]. In addition, amantadine and rimantadine are other antiviruses that target the M2 protein; however, their effectiveness against adamantane-resistant viruses remains to be established. Hence, the development of new lead molecules seems to be crucial, disrupting other processes in the viral life cycle.

In the meantime, the viral RNA polymerase is not yet a target of any approved pharmaceutical. However, its high conservation in strains of avian and human influenza renders it a focus for development of new anti-influenza drugs [25–28]. The RdRp obtains a fundamental role in viral life cycle, but the exact mechanism that develops during it remains poorly understood. It is associated with each viral RNA (vRNA) segment and is involved in both transcription and vRNA replication [29]. 

PB1, PB2, and PA play different roles within polymerase and are all required for both transcription and replication in the nucleus of infected cells. The mass of the heterotrimer of the viral polymerase (P complex) is ~250 kDa. PB1 carries the polymerase active site and an endonuclease activity. It is the core subunit for not only the RNA synthesis but also the assembly of PB2 and PA into this multifunctional enzyme complex. PB1 alone is able to catalyze vRNA-dependent RNA synthesis, but PB2 is responsible for capped RNA-dependent transcription, both together forming the transcriptase. PA and an as yet unidentified host factor(s) are involved in the conversion of RNA polymerase from transcriptase to replicase. 

Despite considerable functional analysis of the RNA polymerase subunits, relatively little is known about their structure [30]. Approaches regarding the whole structural basis of influenza virus (H7N9) RNA polymerase have not been reported, yet. Instead, approaches concerning the structure of different fragments of RNA polymerase subunits have only been made so far. The most significant attempt is associated with the identification of the crystal structure of a fragment of PA of influenza A RNA polymerase that bounds to a fragment of PB1. The carboxy-terminal domain of PA forms a novel fold and a deep, highly hydrophobic groove into which the amino-terminal residues of PB1 can fit by forming a 3(10) helix [26].

Herein, based on a recent phylogenetic analysis [14], we present the three-dimensional *in silico *predicted structures of the H7N9 RNA-dependent RNA polymerase. We focus on the tunnel region on the 3D modelled RdRp, where the oligonucleotide is made, and identify the residues that are key for the function of the enzyme. Strikingly, we found that the 3D conformation of the H7N9 RdRp is very similar to that of the HCV RdRp crystal structure on the molecular level of interaction and bonding. Therefore, we propose that this preliminary study may pave the way for the development of new anti-RdRp agents that may tackle the emerging H7N9 world epidemic.

## 2. Methods

### 2.1. Crystal Structures Used

3D coordinates were obtained from the X-ray solved, crystal structures with RCSB codes: 2W69, 2ZNL, 4F7P, 2YKG, 3A1G, 2ZTT, 4ENF, and 3L56. The methodology that was adopted herein is summarized in the flowchart of Supplementary Figure S1 (see Figure S1 in Supplementary Material available online at http://dx.doi.org/10.1155/2013/645348).

### 2.2. Sequence Alignment

The amino acid sequence of H7N9 viral polymerase was obtained from the GenBank database (accession numbers: AGE08105.1 GI: 444344504 for the PA domain, AGE08108.1 GI: 444344509 for the PB2 domain, and AGE08106.1 GI: 444344506 for the PB1 polymerase domain of the H7N9 viral strain). Using the Gapped-BLAST [31] through NCBI [32], the 2YKG (for the PB1 region) and the 1GTM (for the PB2 region) homologous proteins were identified, which were used as templates for the homology modelling of the H7N9 viral polymerase fragments with no crystallographic structural data. The sequence alignment was done using the online version of ClustalW [33]. The alignment was repeated using hidden Markov models, and the result was the same as the one obtained by ClustalW due to the fact that there are several anchoring conserved motifs throughout the alignment [34].

### 2.3. Homology Modelling

The homology modelling of the H7N9 viral polymerase was carried out using the Modeller package (version 9.10) [35]. The RCSB entries 2YKG (for the PB1 region) and 1GTM (for the PB2 region) were used as templates. The homology model method of Modeller comprises the following steps: firstly, an initial partial geometry specification, where an initial partial geometry for each target sequence is copied from regions of one or more template chains; secondly, the insertions and deletions task, where residues that still have no assigned backbone coordinates are modelled. Those residues may be in loops (insertions in the model with respect to the template), may be outgaps (residues in the model sequence which are aligned before the C-terminus or after the N-terminus of its template), or may be deletions (regions where the template has an insertion with respect to the model). For the purposes of this study outgaps have not been included in the homology modelling process. The third step is the loop selection and side chain packing, where a collection of independent models is created. The last step is the final model selection and refinement one, where the final models are scored and ranked after they have been stereochemically checked with the “Protein Geometry” module for persisting errors. Finally, necessary secondary structure predictions were performed using the NPS (Network Protein Sequence Analysis) web server and the GeneSilico MetaServer which confirmed the choice of the selected template structures for this study.

### 2.4. Molecular Electrostatic Potential (MEP)

Electrostatic potential surfaces were calculated by solving the nonlinear Poisson-Boltzmann equation using finite difference method as implemented in the PyMOL Software [36]. The potential was calculated on grid points per side (65, 65, 65), and the grid filled by solute parameter was set to 80%. The dielectric constants of the solvent and the solute were set to 80.0 and 2.0, respectively. An ionic exclusion radius of 2.0 Å, a solvent radius of 1.4 Å, and a solvent ionic strength of 0.145 M were applied. Amber99 [37] charges and atomic radii were used for this calculation.

### 2.5. Energy Minimization

Energy minimizations were used to remove any residual geometrical strain in each molecular system, using the Charmm27 force field as implemented into the Gromacs suite, version 4.5.5 [38]. All Gromacs-related simulations were performed through our previously developed graphical interface [39]. An implicit Generalized Born (GB) solvation was chosen at this stage, in an attempt to speed up the energy minimization process.

### 2.6. Molecular Dynamics Simulations

Molecular systems were subjected to unrestrained Molecular Dynamics Simulations (MDs) using the Gromacs suite, version 4.5.5 [38]. MDs took place in a SPC water-solvated periodic environment. Water molecules were added using the truncated octahedron box extending 7 Å from each atom. Molecular systems were neutralized with counterions as required. For the purposes of this study, all MDs were performed using the NVT ensemble in a canonical environment at 300 K and a step size equal to 2 femto-seconds for a total of 100 nanoseconds simulation time. An NVT ensemble requires that the number of atoms, volume, and temperature remain constant throughout the simulation. 

### 2.7. Model Evaluation

Evaluation of the model quality and reliability in terms of its 3D structural conformation is very crucial for the viability of this study. Therefore, the produced models were initially evaluated within the Gromacs package by a residue packing quality function, which depends on the number of buried nonpolar side chain groups and on hydrogen bonding. Moreover, the suite PROCHECK [40] was employed to further evaluate the quality of the produced H7N9 influenza virus RdRp model. Verify3D [41] was also used to evaluate whether the model of H7N9 influenza virus RdRp is similar to known protein structures. Finally, the Molecular Operating Environment (MOE) suite was used to evaluate the 3D geometry of the models in terms of their Ramachandran plots, omega torsion profiles, phi/psi angles, planarity, C-beta torsion angles, and rotamer strain energy profiles.

### 2.8. Molecular Docking

In order to *in silico* establish the complex structure of the H7N9 viral polymerase the docking suite ZDOCK (version 3.0) was used [42]. Docking experiments were conducted on the models that had been energetically minimized and conformationally optimized using molecular dynamics simulations. ZDOCK is a protein-protein docking suite that utilizes a grid-based representation of the molecular system involved. In order to efficiently explore the search space and docking positions of the molecules as rigid bodies, ZDOCK takes full advantage of a three-dimensional fast Fourier transformation algorithm. It uses a scoring function that returns electrostatic, hydrophobic, and desolvation energies as well as performing a fast pairwise shape complementarity evaluation. Moreover, it uses the contact propensities of transient complexes of proteins to perform an evaluation of a pairwise atomic statistical potential for the docking molecular system. RDOCK was utilized to refine and quickly evaluate the results obtained by ZDOCK [42]. RDOCK performs a fast minimization step to the ZDOCK molecular complex outputs and reranks them according to their recalculated binding free energies.

## 3. Results/Discussion

The RNA-dependent RNA polymerase of the H7N9 viral strain is comprised of the separate domains, namely, the domains PA, PB1, and PB2. For the purposes of this study, a combination of molecular modelling techniques was employed in an effort to model the full complex structure of the H7N9 strain. The resulting model was then evaluated for its accuracy and viability using both a series of *in silico *tools and a direct comparison to the X-ray crystal structure of the Hepatitis C virus RNA-dependent RNA polymerase. Strikingly, it was found that the H7N9 RdRp shared a similar substrate interaction pattern with the Hepatitis C RdRp crystal structure.

The PA subunit plays differential roles; it induces a generalized proteolysis and an endonucleolytic processing [43], binds to the vRNA and cRNA promoters [44], and interacts with PB1 subunit [26]. Crystallization of PA and PB1 N-terminal complex indicated that catalytic residues of endonuclease active site [45] are conserved among influenza A strains and are found in N-terminal PA domain (PA_N_ residues 1–197 [46]). Specifically, they are comprised of His41, Glu80, Asp108, and Glu119. Subsequently, several attempts of developing anti-influenza drugs were performed [47], but none of them turned out to be really effective. However, potency and specificity improvement in 3D structure may enable chemotherapeutic agents, that mimic the PA_N_ active site, to be novel potential inhibitors [46]. 

The RdRp active site is included in PB1 subunit. PA_C_ and PB1_N_ interactions seem to obtain a crucial role in both viral transcription and replication. Only few residues (2–15) of PB1_N_ bind PA_C_. They are highly conserved in several influenza A viruses and are responsible for the complex stability. PB1 also interacts with PB2 N-terminal domain. Specifically, three helices from each of the domains PB1_C_ (residues 678–757) and PB2_N_ (residues 1–35) are bundled to form a “revolver-shaped” structure [48]. 

Regarding further PB2 functionality, crystal structures indicated that residues from 318 to 483 are responsible for cap-binding [49]. In the meantime, the similar cap-binding mode of the host cap-binding proteins renders anti-influenza drug development as a real challenge [50].

In addition, NMR and crystallization [51, 52], performed techniques on PB2_c_, revealed that a nuclear localization signal (NLS) sequence [53] is responsible for nuclear import from the cytoplasm. The bipartite NLS sequence is located in PB2_c_ (residues 678–757). Moreover, there is a C-terminal fragment [54] near the NLS sequence containing the residue Lys627 [51], which seems to be involved in viral replication. However, the exact mechanism of viral replication has not been elucidated, yet. So far, the absence of a complete structure of the RdRp complex fails to explain molecular functionality. 

### 3.1. Homology Modelling

The sequence alignment and the blastp query that followed revealed that the PA domain was available in the PDB databank. On the other hand, only a small region of the PB1 and a relatively larger region of the PB2 domain either were available as crystal structures or could be modelled using homology-based molecular modelling techniques. The PA domain was established by the docking of an influenza polymerase fragment (RCSB entry: 2W69) and the crystal structure of the PA-PB1 complex form of the influenza virus RNA polymerase (RCSB entry: 2ZNL). The total cover of the PA sequence alignment exceeds 90%, while the percentage identity and similarity reach 90% and 93%, respectively (Supplementary Material 1). The PB1 domain was only partially modelled by combining two crystal structures and a homology-build model. More specifically, the B chain of the crystal structure of the PA-PB1 complex form of the influenza virus RNA polymerase (RCSB entry: 2ZNL) was docked with the C chain of the crystal structure of the HLA-A2402 (RCSB entry: 4F7P) and the A chain of the crystal structure of the PB1-PB2 subunits from influenza A virus (RCSB entry: 3A1G). The final docking component was the partial 3D model of the PB1 region with the template structure of the A chain of the crystal structure of the RNA recognition by Rig-I protein (RCSB entry: 2YKG) (Supplementary Material 2). Even though the sequence identity was found to be quite low (22%), the sequence similarity for that region was high enough (48%) to allow conventional homology modelling to be considered. Likewise, the assembly of the PB2 domain involved the iterative docking of the B chain of the crystal structure of the PB1-PB2 subunits from influenza A virus (RCSB entry: 2ZTT), the crystal structure of the cap-binding domain of the polymerase basic protein 2 from influenza A virus (RCSB entry: 4ENF), and the crystal structure of the large C-terminal domain of the polymerase basic protein 2 from influenza A virus (RCSB entry: 3L56). The region of the PB2 domain that was modelled shared 21% of sequence identity and 47% of sequence similarity with its template structure of the glutamate dehydrogenase protein (RCSB entry: 1GTM) (Supplementary Material 3). The selection of the most suitable template was achieved using a combination of blastp searches and fold recognition tools. 

More specifically, protein fold recognition techniques (FR) aim to identify and pinpoint similarities among 3D protein structures that are not supplemented by significant sequence similarity. The underlying principle behind FR techniques is that a quick search for protein folds is made in large protein databases, which is looking to identify folds that are compatible with a particular sequence. Unlike simple comparisons based on sequence only, these more sophisticated methods exploit all the extra 3D structural information that is readily available for many proteins. In essence these techniques turn the protein folding problem around: rather than predicting how a sequence will fold, they predict how well a fold will fit a sequence [52]. Both H7N9 RdRp homology models constitute one of these striking examples of structurally and functionally identical enzymes, which only share a low primary sequence identity. 

The first structural superimposition between the H7N9 RdRp PB1 model and its template exhibited an alpha-carbon RMSD that falls well within 0,84 angstroms. The H7N9 RdRp PB1 model was consequently checked with PROCHECK for its geometry mathematical accuracy. In addition to that, the Verify3D algorithm was employed for a more in-depth evaluation for its structure. A direct compatibility comparison between the H7N9 RdRp PB1 model to its own amino acid sequence was performed by Verify3D. Judging strictly on location and environment, each residue is assigned a structural class. In order to do this, a rather large database of reference structures is being used as a control. The H7N9 RdRp PB1 model scored a very reliable range between +0.12 and +0.33. This was a further confirmation in that the established H7N9 RdRp PB1 model is of high quality and mathematically reliable. Verify3D scores that fall below the +0.1 mark are indicative of major problems in the structure of the model, as it can be mathematically evaluated [41]. A summary of the output of the various geometrical assessment tools that were used can be found in Supplementary Figure S2.

### 3.2. Molecular Docking

In an effort to construct the full 3D structure of the H7N9 RdRp protein the ZDOCK algorithm was employed. The docking was conducted in a pairwise function until each one of the three H7N9 RdRp domains was established. More specifically, the 3D model of the PA domain of the H7N9 RdRp protein was established with the molecular docking of the 2W69 and 2ZNL X-ray crystal structures. Likewise, the 3D model of the PB1 domain of the H7N9 RdRp protein was established by the molecular docking of the 4F7P fragment on the relevant region of the 2ZNL X-ray crystal structure. Note that the 2ZNL crystal structure contains fragments of both the PA and PB1 regions. Then, iteratively, 3A1G structure was docked on the latter complex, and finally, the PB1 homology-build fragment was added too by molecular docking to the latter three-mere complex. The PB2 structure was obtained by the molecular docking of the 3L56 and the 4ENF crystal structures. In a following docking steps the 2ZTT crystal structure was added, and finally, the PB2 homology-build fragment was added too. 

**Figure 1:**
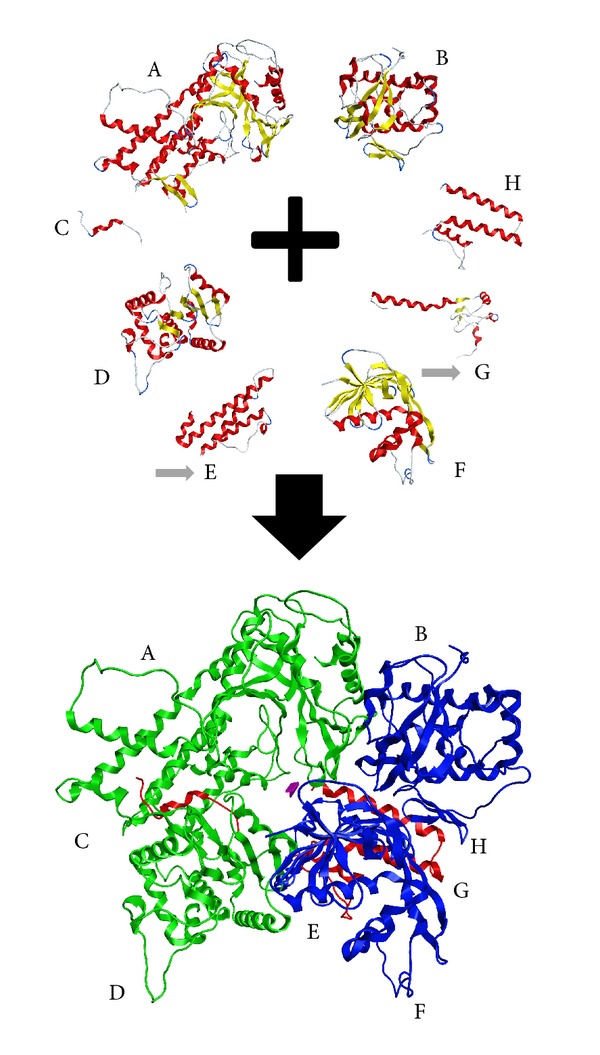
The homology modelling and docking experiment for the establishment of the 3D structure of the H7N9 RdRp structure. Top: the components that were used to construct the 3D complex model of the H7N9 RdRp. A, B, C, D, F, and H are crystal structures, whereas E and G (pointed by the grey arrows) have been built using homology-modelling techniques. Bottom: the full complex structure of the H7N9 RdRp as established by the docking experiment. The PA domain is shown in green, the PB1 in blue, and the pB2 in red ribbon representation.

The establishment of the full H7N9 RdRp heterotrimer protein was established by the iterative 3D molecular docking of the PA and PB1 domains first. Then the previously established PB2 domain was docked on the PA-PB1 docked complex (Figure 1).

### 3.3. Interaction Patterns with the Substrate in the H7N9 RdRp Model

**Figure 2:**
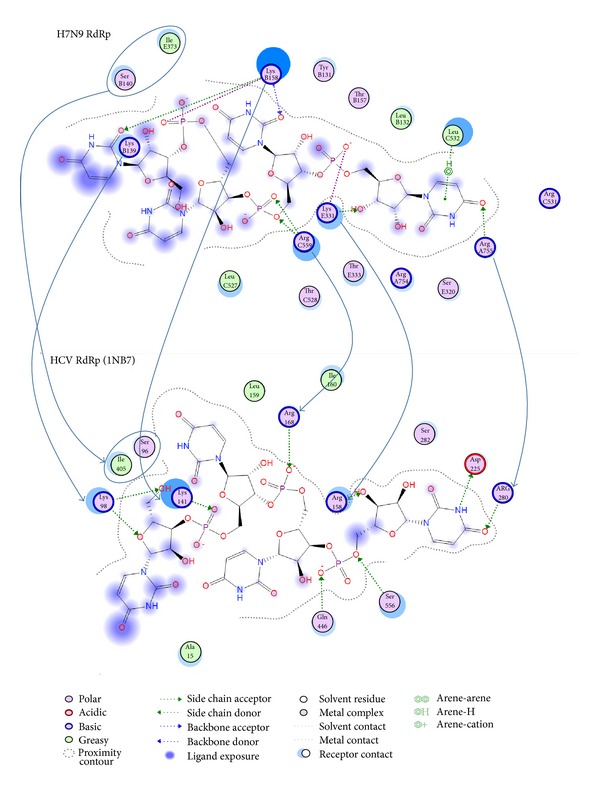
Molecular interaction maps of the H7N9 and HCV RdRp proteins. Top: the H7N9 RdRp interaction pattern with the docked oligonucleotide. Bottom: the HCV RdRp (RCSB entry: 1NB7) interaction pattern with the cocrystallized oligonucleotide. The corresponding and structurally conserved residues are shown with the blue arrows.

**Figure 3:**
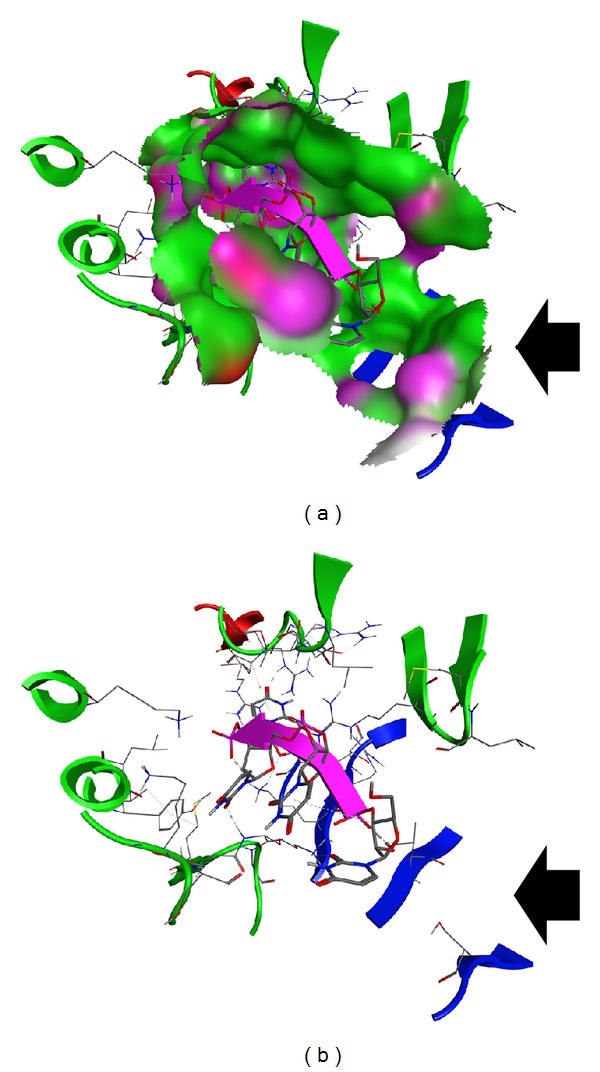
The 3D spatial conformation of the H7N9 RdRp oligonucleotide tunnel. (a) The shape of the H7N9 RdRp oligonucleotide tunnel. The ssRNA fragment is shown in purple color. (b) The residues that comprise the active sites of the H7N9 RdRp oligonucleotide tunnel. The two “roadblocking” serine and isoleucine residues are indicated by the large black arrows. Their role is to prevent the oligonucleotide to reversely slide out in the wrong direction.

In an effort to confirm the functionality, suitability, and reliability of the H7N9 RdRp model to be used in structure-based drug design experiments, the specific interactions with its ssRNA substrate should be determined. In this direction the cocrystallized oligonucleotide fragment from the HCV RdRp was borrowed and was consecutively docked into our H7N9 RdRp model. The molecular docking experiment was followed by exhaustive molecular dynamics simulations (MDs). MDs were performed to the H7N9 RdRp model in the presence of the oligonucleotide substrate, in an explicitly solvated periodic box with SPC water molecules (energy versus time plot in Supplementary Figure S3). Strikingly, postmolecular dynamics analysis revealed that the top-ranked (lowest molecular system energy) oligonucleotide pose does not differ much from the HCV mode of interaction in the crystal structure (Figure 2). More specifically, there is a network of conserved amino acids and molecular interactions between the H7N9 RdRp model and the HCV RdRp crystal structure. The nucleotide at the –OH end is stabilized by hydrogen bonding to an Arginine residue both in the H7N9 RdRp model and the HCV RdRp structure. Arg755:A establishes a hydrogen bond with the =O group of the –OH prime base. The ring is stabilized by hydrophobic interactions to Leu532:C. The corresponding residues on the HCV RdRp are the Arg230 and the Asp225 residues. The Asp225 on the HCV RdRp establishes a hydrogen bond with the –NH group of the phenyl ring of the –OH base, which, however, serves the same purpose with the Leu532:A hydrophobic interaction in H7N9 RdRp model. One of the –OH groups of the first substrate nucleotide sugar moiety establishes hydrogen bond with a lysine amino acid (Lys331:E) in the H7N9 RdRp model, whereas it is an Arginine residue (Arg158) in the HCV RdRp crystal structure. The rest of the oligonucleotide interactions involve a set of two lysine residues in both the H7N9 RdRp model and the HCV RdRp crystal structure. These are the Lys158:B and Lys139:B in the H7N9 RdRp model, while the corresponding ones in the HCV RdRp crystal structure are the Lys141 and Lys98. Finally, it was found that the inner part of the oligonucleotide tunnel is blocked by a serine and an isoleucine residue in both H7N9 RdRp model and HCV RdRp crystal structure. We assume that these residues act as a flexible roadblock that stops the oligonucleotide to move towards the wrong direction in the RdRp tunnel. The relative positioning of these two residues on the H7N9 RdRp model is shown in Figure 3 (black arrow).

### 3.4. Electrostatic Potential Surfaces

**Figure 4:**
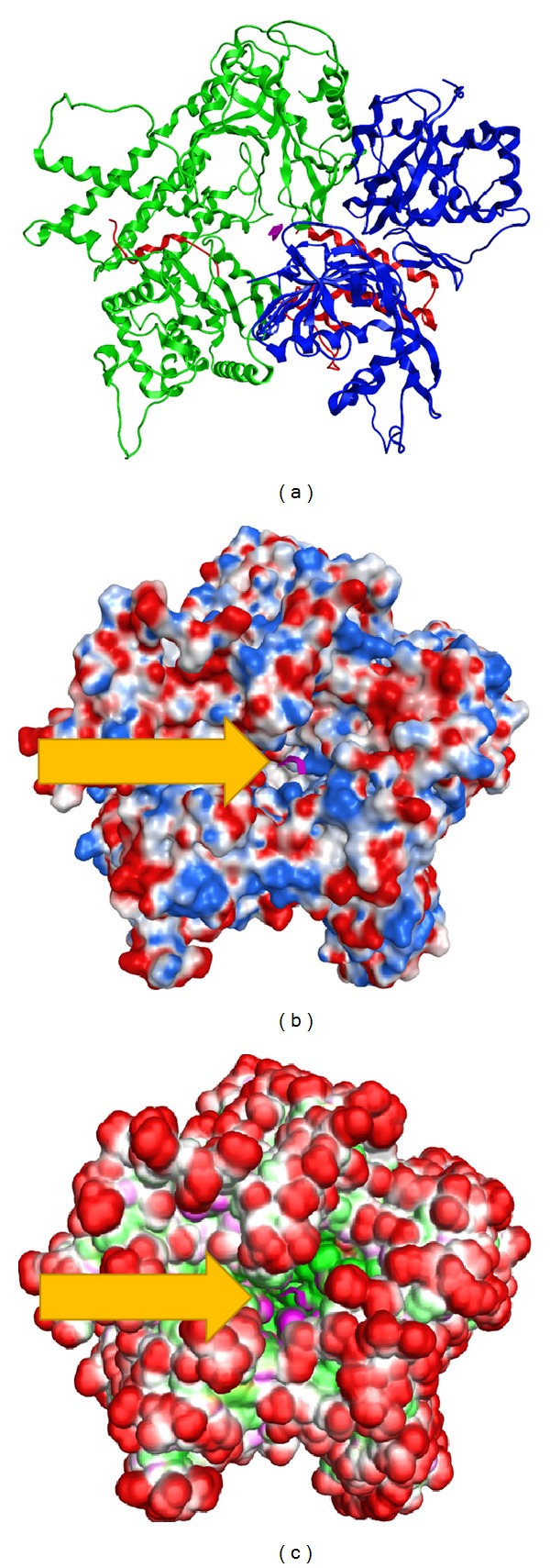
Molecular surfaces of the H7N9 RdRp protein. (a) The 3D model of the H7N9 RdRp complex. (b) The electrostatic surface of the H7N9 RdRp. (c) The ActiveLP (size, shape, and hydrophobic) representation of the H7N9 RdRp protein. The oligonucleotide in its tunnel is shown in purple color and is indicated by the yellow arrows.

The molecular surface of the produced H7N9 RdRp model was analyzed by calculating its electrostatic potential and 3D spatial anatomy (Figure 4(b)). It was found that the oligonucleotide tunnel in the H7N9 RdRp model mainly consists of positively charged residues, as is the case with most viral RdRp proteins. The main purpose of this high positive local charge is to attract the negatively charged oligonucleotide backbone. The charges on the rest of the H7N9 RdRp model are evenly distributed. This finding is in perfect agreement with the HCV structure that was previously compared with our model. The HCV RdRp crystal structure shares a positively charged entrance to its oligonucleotide tunnel. Finally, the ActiveLP surface was calculated for the H7N9 RdRp model (Figure 4(c)). Active LP colors the surface to indicate hydrophobic regions, mildly polar regions, and hydrogen bonding regions taking directionality into account. In essence, this surface representation colors the surface so that “deep pocket” and solvent exposed regions are highlighted. It was found that the H7N9 RdRp model exhibits a pretty protected hydrophilic outer cell, while the inner part is pretty hydrophobic and anatomically designed to receive the nucleotide chain (Figure 4, purple color).

### 3.5. Conclusions and Insights Obtained from the H7N9 RdRp Model

The 3D model of the H7N9 influenza A strain RdRp was designed using a combination of 3D molecular modelling techniques. A series of crystal structures were used alongside two homology-built models for the parts of the protein without known structure. The final 3D complex of the H7N9 RdRp model was established using molecular docking and molecular dynamics simulations. Strikingly, it was found that the oligonucleotide tunnel in the H7N9 RdRp model shares high similarity in terms of its 3D spatial arrangement and amino acid composition with the HCV RdRp tunnel. The structure of the HCV RdRp has long ago been crystallized and extensively studied. However this is not the case with the H7N9 RdRp model. Herein, we conclude that since the 3D structures of the oligonucleotide tunnels of the two proteins are so similar, knowledge from the HCV RdRp research could be used against the H7N9 RdRp model too. Finally, the “key” residues in the catalytic site of the H7N9 RdRp model have been identified, alongside the 3D anatomical unique characteristics of the latter enzyme, in an effort to provide insights for future structure-based drug design or virtual high throughput experiments, which may lead to the establishment of a well-supported antiviral strategy against the H7N9 lethal strain of the influenza A that is currently in epidemic in many places of the world.

## Supplementary Material

This is a seven step approach that begins with the sequence alignment and ends with the analysis of the produced 3D in silico models.

## Abbreviations

FR: Fold recognition
GB: Generalized born
HA: Hemagglutinin
MDs: Molecular dynamics simulations
MOE: Molecular operating environment
M1: Matrix 1
M2: Matrix 2
NA: Neuraminidase
NP: Nucleoprotein
NSP1: Nonstructural protein 1
NS2: Nonstructural protein 2
PA: Polymerase acidic protein
PB1: Polymerase basic protein 1
PB1-F2: Polymerase basic protein 1-F2
PB2: Polymerase basic protein 2
RdRp: RNA-dependent RNA polymerase
ssRNA: Single-stranded RNA
vRNA: Viral RNA
vRNPs: Viral ribonucleoproteins
WHO: World Health Organization.
