# Supplementary material for: 3D Molecular Modelling Study of the H7N9 RNA-Dependent RNA Polymerase as an Emerging Pharmacological Target
Source: Influenza Res Treat. 2013 Sep 25;2013:645348. doi: 10.1155/2013/645348 (PMC3800656; doi:10.1155/2013/645348)
Supplement: Supplementary file 1 — This is a seven step approach that begins with the sequence alignment and ends with the analysis of the produced 3D in silico models. [file 645348.f1.pdf]

Supplementary Material 1

<u>PA Full Alignment</u>

(CRYSTAL STRUCTURE: RCSB entry: 2W69)

```
Query    MEDFVRQCFNPMIVELAEKAMKEYGEDPKIETNKFAAICTHLEVCFMYSDFHFIDERGES  60
         MEDFVRQCFNPMIVELAEKAMKEYGED KIETNKFAAICTHLEVCFMYSDFHFI+E+GES
Sbjct    MEDFVRQCFNPMIVELAEKAMKEYGEDLKIETNKFAAICTHLEVCFMYSDFHFINEQGES  67

Query    IIVESGDPNALLKHRFEIIEGRDRTMAWTVVNSICNTTGVEKPKFLPDLYDYKENRFIEI  120
         I+VE  DPNALLKHRFEIIEGRDRTMAWTVVNSICNTTG EKPKFLPDLYDYKENRFIEI
Sbjct    IVVELDDPNALLKHRFEIIEGRDRTMAWTVVNSICNTTGAEKPKFLPDLYDYKENRFIEI  127

Query    GVTRREVHIYYLEKANKIKSEKTHIHIFSFTGEEMATKADYTLDEESRARIKTRLFTIRQ  180
         GVTRREVHIYYLEKANKIKSE THIHIFSFTGEEMATKADYTLDEESRARIKTRLFTIRQ
Sbjct    GVTRREVHIYYLEKANKIKSENTHIHIFSFTGEEMATKADYTLDEESRARIKTRLFTIRQ  187

Query    EMANRGLWDSFRQSERGEETIEERFEITG  209
         EMANRGLWDSFRQSERGEETIEERFEITG
Sbjct    EMANRGLWDSFRQSERGEETIEERFEITG  216
```

…

(CRYSTAL STRUCTURE: RCSB entry: 2ZNL)

```
Query    NGCIEGKLSQMSKEVNARIEPFLKTTPRPLRLPEGPPCSQRSKFLLMDALKLSIEDPSHE  298
         NG IEGKLSQMSKEVNARIEPFLKTTPRPLRLP GPPCSQRSKFLLMDALKLSIEDPSHE
Sbjct    NGYIEGKLSQMSKEVNARIEPFLKTTPRPLRLPNGPPCSQRSKFLLMDALKLSIEDPSHE  60

Query    GEGIPLYDAIKCMKTFFGWKEPNIVKPHEKGINPNYLLAWKQVLAELQDIENEEKIPKTK  358
         GEGIPLYDAIKCM+TFFGWKEPN+VKPHEKGINPNYLL+WKQVLAELQDIENEEKIPKTK
Sbjct    GEGIPLYDAIKCMRTFFGWKEPNVVKPHEKGINPNYLLSWKQVLAELQDIENEEKIPKTK  120

Query    NMKKTSQLKWALGENMAPEKVDFEDCKDVSDLRQYDSDEPEQRSLASWIQSEFNKACELT  418
         NMKKTSQLKWALGENMAPEKVDF+DCKDV DL+QYDSDEPE RSLASWIQ+EFNKACELT
Sbjct    NMKKTSQLKWALGENMAPEKVDFDDCKDVGDLKQYDSDEPELRSLASWIQNEFNKACELT  180

Query    DSSWIELDEIGEDVAPIEHIASMRRNYFTAEVSHCRATEYIMKGVYINTALLNASCAAMD  478
         DSSWIELDEIGEDVAPIEHIASMRRNYFT+EVSHCRATEYIMKGVYINTALLNASCAAMD
Sbjct    DSSWIELDEIGEDVAPIEHIASMRRNYFTSEVSHCRATEYIMKGVYINTALLNASCAAMD  240

Query    DFQLIPMISKCRTKEGRRKTNLYGFIIKGRSHLRNDTDVVNFVSMEFSLTDPRLEPHKWE  538
         DFQLIPMISKCRTKEGRRKTNLYGFIIKGRSHLRNDTDVVNFVSMEFSLTDPRLEPHKWE
Sbjct    DFQLIPMISKCRTKEGRRKTNLYGFIIKGRSHLRNDTDVVNFVSMEFSLTDPRLEPHKWE  300

Query    KYCVLEIGDMLLRTAIGQVSRPMFLYVRTNGTSKIKMKWGMEMRRCLLQSLQQIESMIEA  598
         KYCVLEIGDML+R+AIGQVSRPMFLYVRTNGTSKIKMKWGMEMRRCLLQSLQQIESMIEA
Sbjct    KYCVLEIGDMLIRSAIGQVSRPMFLYVRTNGTSKIKMKWGMEMRRCLLQSLQQIESMIEA  360

Query    ESSVKEKDMTREFFENKSETWPIGESPKGVEEGSIGKVCRTLLAKSVFNSLYASPQLEGF  658
         ESSVKEKDMT+EFFENKSETWPIGESPKGVEE SIGKVCRTLLAKSVFNSLYASPQLEGF
Sbjct    ESSVKEKDMTKEFFENKSETWPIGESPKGVEESSIGKVCRTLLAKSVFNSLYASPQLEGF  420

Query    SAESRKLLLIVQALRDNLEPGTFDLGGLYEAIEECLINDPWVLLNASWFNSFLTHAL  715
         SAESRKLLLIVQALRDNLEPGTFDLGGLYEAIEECLINDPWVLLNASWFNSFLTHAL
Sbjct    SAESRKLLLIVQALRDNLEPGTFDLGGLYEAIEECLINDPWVLLNASWFNSFLTHAL  477
```

Supplementary Material 2

PB1 Full Alignment

(CRYSTAL STRUCTURE: RCSB entry: 2ZNL)

```
Query     MDVNPTLLFLKVPAQNAISTTFPYTGDPPYSHGTGTGYTMDTVNRTHQYSEKGKWTTNSE  60
            MDVNPTLLFLKVPAQNAISTTFPYTGDPPYSHGTGTGYTMDTVNRTHQYSEKG+WTTN+E
Sbjct     MDVNPTLLFLKVPAQNAISTTFPYTGDPPYSHGTGTGYTMDTVNRTHQYSEKGRWTTNTE  60

Query     TGAPQLNPIDGPLPEDNEPSG  81
            TGAPQLNPIDGPLPEDNEPSG
Sbjct     TGAPQLNPIDGPLPEDNEPSG  81
```

…

(CRYSTAL STRUCTURE: RCSB entry: 4F7P)

```
Query     FYRYGFVANF  505
            FYRYGFVANF
Sbjct     FYRYGFVANF  10
```

…

(HOMOLOGY MODEL #1 ALINGMENT. TEMPLATE:2YKG)

```
Query     DTQIQTRRSFELKKLWEQTRSKAGLLVSDGGPNLYNIRN------------LHIPEVCLK  612
             +++I  +  + + +L   T S A  +  D   NL  I+N            + + + C+
Sbjct     ESRISDKFKYIIAQLMRDTESLAKRICKDL-ENLSQIQNREFGTQKYEQWIVTVQKACMV  293

Query     WELMDEDYQGRLCNPLNPFVSHKEIESVNNAVVMPAHGPAK-SMEY  657
           +++ D+D + R+C  L  + SH  +   N+A+++  H  K +++Y
Sbjct     FQMPDKDEESRICKALFLYTSH--LRKYNDALIISEHARMKDALDY  337
```

…

(CRYSTAL STRUCTURE: RCSB entry: 3A1G)

```
Query     SQRGILEDEQMYQKCCNLFEKFFPSSSYRRPVGISSMVEAMVSRARIDARIDFESGRIKK  737
            SQRG+LEDEQ YQ+CCNLFEKFFPSSSYRRPVGISS VEA VSRARIDARIDFESGRIKK
Sbjct     SQRGVLEDEQXYQRCCNLFEKFFPSSSYRRPVGISSXVEAXVSRARIDARIDFESGRIKK  60

Query     EEFAEIMKICSTIEELRRQK  757
            EEF EI KICSTIEELRRQK
Sbjct     EEFTEIXKICSTIEELRRQK  80
```

Supplementary Material 3

PB2 Full Alignment

(CRYSTAL STRUCTURE: RCSB entry: 2ZTT)

```
Query  2   ERIKELRDLMSQSRTREILTKTTVDHMAIIKKYTSG  37
           ERIKELR+L SQSRTREILTKTTVDH AIIKKYTSG
Sbjct  5   ERIKELRNLXSQSRTREILTKTTVDHXAIIKKYTSG  40
```

…

(HOMOLOGY MODEL #2 ALINGMENT. TEMPLATE:1GTM)

```
Query      PGHADLSAKEAQDVIMEVVFPNEVGARILTSESQLTITKEKKEELQDCKIAPLMVAYMLE  208
           PG  +++ +E  ++ ++V+ P  +    ++T ++   I  +   E+ +  + P     + E
Sbjct      PGATNITNEELLELEVDVLAPAAI-EEVITKKNADNIKAKIVAEVANGPVTPEADEILFE  332

Query      RELVRKTRFLPVAGGTSSVYIEVLHLTQGTCW  240
            + +++   FL  AGG +  Y E +    G  W
Sbjct      KGILQIPDFLCNAGGVTVSYFEWVQNITGYYW  364
```

…

(CRYSTAL STRUCTURE: RCSB entry: 4ENF)

```
Query      LRISSSFSFGGFTFKRTSGSSVKREEEVLTGNLQTLKIRVHEGYEEFTMVGRRATAILRK  376
           +RISSSFSFGGFTFKRTSGSSVKREEEVLTGNLQTLKIRVHEGYEEFTMVGRRATAILRK
Sbjct      MRISSSFSFGGFTFKRTSGSSVKREEEVLTGNLQTLKIRVHEGYEEFTMVGRRATAILRK  63

Query      ATRRLIQLIVSGRDEQSIAEAIIVAMVFSQEDCMIKAVRGDLNFVNRANQRLNPMHQLLR  436
           ATRRLIQLIVSGRDEQSIAEAIIVAMVFSQEDCMIKAVRGDLNFVNRANQRLNPMHQLLR
Sbjct      ATRRLIQLIVSGRDEQSIAEAIIVAMVFSQEDCMIKAVRGDLNFVNRANQRLNPMHQLLR  123

Query      HFQKDAKVLFQNWGIEPIDNVMGMIGILPDMTPSTEMSLRGIRVSKM  483
           HFQKDAKVLFQNWG+EPIDNVMGMIGILPDMTPS EMS+RG+R+SKM
Sbjct      HFQKDAKVLFQNWGVEPIDNVMGMIGILPDMTPSIEMSMRGVRISKM  170
```

…

(CRYSTAL STRUCTURE: RCSB entry: 3L56)

```
Query      EINGPESVLVNTYQWIIRNWETVKIQWSQDPTMLYNKMEFEPFQSLVPKAARGQYSGFVR  597
           EINGPESVLVNTYQWIIRNWETVKIQWSQDPTMLYNKMEFEPFQSLVPKAARGQYSGFVR
Sbjct      EINGPESVLVNTYQWIIRNWETVKIQWSQDPTMLYNKMEFEPFQSLVPKAARGQYSGFVR  60

Query      TLFQQMRDVLGTFDTVQIIKLLPFAAAPPEQSRMQFSSLTVNVRGSGMRILVRGNSPVFN  657
           TLFQQMRDVLGTFDTVQIIKLLPFAAAPP+QSRMQFSSLTVNVRGSGMRILVRGNSPVFN
Sbjct      TLFQQMRDVLGTFDTVQIIKLLPFAAAPPKQSRMQFSSLTVNVRGSGMRILVRGNSPVFN  120

Query      YTKTTKRLTVLGKDAGALTEDPDEGTAGVESAVLRGFLILGKEDKRYGPALSINELSNLA  717
           Y K TKRLTVLGKDAGALTEDPDEGTAGVESAVLRGFLILGKEDKRYGPALSINELSNLA
Sbjct      YNKATKRLTVLGKDAGALTEDPDEGTAGVESAVLRGFLILGKEDKRYGPALSINELSNLA  180

Query      KGEKANVLIGQGDVVLVMKRKRDSSILTDSQTATKRIRMAIN  759
           KGEKANVLIGQGDVVLVMKRKRDSSILTDSQTATKRIRMAIN
Sbjct      KGEKANVLIGQGDVVLVMKRKRDSSILTDSQTATKRIRMAIN  222
```
